# How Did the COVID-19 Pandemic Effect Dental Patients? An Italian Observational Survey Study

**DOI:** 10.3390/healthcare9121748

**Published:** 2021-12-17

**Authors:** Gianna Maria Nardi, Roberta Grassi, Felice Roberto Grassi, Roberto Di Giorgio, Fabrizio Guerra, Livia Ottolenghi, Giovanna Acito, Nasrin Basari, Simone Bisegna, Lorella Chiavistelli, Roberta Cimarossa, Arcangela Colavito, Luigina Figlia, Claudio Gabrielli, Silvia Sabatini, Maciej Jedliński, Marta Mazur

**Affiliations:** 1 Department of Oral and Maxillofacial Sciences, Sapienza University of Rome, 00161 Rome, Italy; giannamaria.nardi@uniroma1.it (G.M.N.); roberto.digiorgio@uniroma1.it (R.D.G.); fabrizio.guerra@uniroma1.it (F.G.); livia.ottolenghi@uniroma1.it (L.O.); marta.mazur@uniroma1.it (M.M.); 2 Department of Biomedical Sciences, University of Sassari, 07100 Sassari, Italy; grassi.roberta93@gmail.com; 3 Department of Basic Medical Sciences, Neurosciences and Sense Organs, Aldo Moro University of Bari, 70122 Bari, Italy; feliceroberto.grassi@uniba.it; 4 ATASIO: Accademia delle Tecnologie Avanzate nelle Scienze di Igiene Orale-Academy of Advanced Technologies in Oral Hygiene Sciences, 70126 Bari, Italy; giovanna.acito@unimore.it (G.A.); nasrinhb@yahoo.it (N.B.); simone.bisegna90@libero.it (S.B.); lorella.chiavistelli@gmail.com (L.C.); roby.cimarossa@gmail.com (R.C.); dottsa.colavito@gmail.com (A.C.); lu.figlia@libero.it (L.F.); claudio.gabrielli.contacts@gmail.com (C.G.); silvia.sabatini@unimore.it (S.S.); 5 Department of Interdisciplinary Dentistry, Pomeranian Medical University in Szczecin, 70-111 Szczecin, Poland

**Keywords:** COVID-19, dental patients, anxiety, health behaviors, oral hygiene

## Abstract

The primary aim of this observational survey study was to assess patients’ attitudes toward clinical dental practice during the COVID-19 pandemic; the secondary aim was to evaluate patients’ attitudes towards oral health by maintaining an appropriate lifestyle and oral hygiene at home. The questionnaire was developed using Google Forms. The questionnaire consisted of three parts: Part A—geographic, demographic, and personal data; Part B—patients’ attitude toward oral health selfcare and lifestyle; Part C—patients’ attitude toward dental practice. This survey, conducted during the months of November and December 2020, enrolled 1135 subjects throughout Italy. All data were statistically analyzed. COVID-19 has changed patients’ approach to dental procedures. Most of the people interviewed lived in families, and their greatest fear was infecting a family member. Restrictive measures forced people to stay at home, which led to an increased consumption of various types of food, including cariogenic foods. People said they felt safe when they went to the dentist, but they also paid special attention to measures to prevent contagion. Among the measures that should be introduced in similar situations in the future, people wanted telemedicine, a phone recall, and the possible use of video clips for home oral care instructions.

## 1. Introduction

In December 2019, the novel coronavirus (SARS-CoV-2) was identified in China; soon after, in February 2020, the virus spread to Europe, and finally, on 11 March 2020, the World Health Organization called it COVID-19 and declared it a pandemic [1].

By 25 March 2020, the country with the most confirmed COVID-19 cases was the United States, with 81,864 cases, and Italy was the most affected country in number of deaths, with 74,386 cases and 7503 deaths [1].

Health systems’ capabilities and performance were the main issues in all the affected countries, and in the interest of “flattening the curve” [2], meaning to decrease the number of new cases and hence the number of patients requiring hospitalization—restrictive measures were put in place. The restrictions consisted in flight limitation, borders closing, shutting down cafes and restaurants, closing of schools, and self-isolation at first and restriction of movement afterwards, with a total lockdown being the last resort [2].

In this scenario, dental practice was identified as a working place with a most potential high risk of cross-infection, as face-to-face communication and consistent exposure to body fluids, such as blood and saliva, are frequent and common in dentistry [3].

COVID-19 transmission in dental settings was described, and it was assessed that it occurs through four major routes: (1) direct exposure to respiratory secretions containing droplets, blood, saliva, or other patient materials; (2) indirect contact with contaminated surfaces and/or instruments; (3) inhalation of suspended airborne viruses; and (4) mucosal (nasal, oral, and conjunctival) contact with infection-containing droplets and aerosols that are produced by dental procedures [4,5].

Control of infection transmission in dental offices was achieved through the use of well-defined protocols and operating procedures, with a strong recommendation for all the healthcare personnel to use personal protective equipment, such as masks, protective goggles, gowns, helmet, gloves, caps, face shields, and shoe covers. In the meantime, dental patients were also allowed to be treated with special considerations when treatment was urgent and could not be postponed. The combined use of mouth rinses, rubber dam, anti-retraction handpieces, and disinfectants proved to lower the risk of COVID-19 transmission in the dental setting [6].

The primary aim of this observational survey study was to assess patients’ attitudes toward clinical dental practice during the COVID-19 pandemic; the secondary aim was to evaluate patients’ attitudes towards oral health by maintaining an appropriate lifestyle and oral hygiene at home.

## 2. Materials and Methods

### 2.1. Study Type

An observational survey study was carried out. The study was approved by the Institutional Review Board of territorial NHS facilities (n. 080420). The survey was carried out from 1 November to 30 December 2020.

### 2.2. Study Population

Patients in dental clinical settings throughout Italy were asked to answer to the questionnaire.

### 2.3. Data Collection Technique

A team of 10 hygienists belonging to the AtasioLab Research Club completed the questionnaire with patients in dental clinical settings throughout Italy. The questionnaire was developed using Google Forms (Google, Mountain View, CA, USA) tool.

The questionnaire consisted of three parts: Part A—geographic, demographic, and personal data (questions *n* = 10); Part B—respondents’ attitude toward oral health selfcare and lifestyle (*n* = 16); Part C—respondents’ attitude toward dental practice (*n* = 13).

### 2.4. Data Analysis Procedure

The R statistical program [7] was used for the calculations and graphical presentation of results. The variables in regression models were considered statistically significant at *p* < 0.05, and model specifications were chosen by Akaike Information Criterion (AIC) [8].

## 3. Results

### 3.1. Part A—Geographic, Demographic, and Personal Data (Questions n = 10)

A total of 1135 subjects participated into the study. Female participants were 782 (68.9%) and male 353 (31.1%). Age ranges were 14–20, 3.0%; 20–40, 51.8%; 40–60, 38.2%; and 60–80, 7%. Area of geographic origin was 54.1% in the Center of Italy and 20.6% and 25.3% in the north and the south, respectively. The majority of the participants were married (63.9%). Single people were 25.9%, in separation 8.6%, and widow(er)s were 1.6%. The vast majority of 80.2% lived with family, while 19.8% lived alone; of these, 45.8% had children, and 54.2% did not. The level of education was basic, secondary, and higher for 6%, 47%, and 46.7% of the respondents, respectively. The major social characteristics of respondents are summarized in Table 1.

### 3.2. Part B—Patients’ Attitude toward Oral Health Selfcare and Lifestyle (n = 16)

In the second part of the questionnaire the patient’s responses were collected regarding the frequency of professional oral hygiene sessions before and during the COVID-19 pandemic, changes in the direction of professional and at-home oral procedures during the COVID-19 pandemic, at-home oral hygiene habits during COVID-AP, and lifestyle changes, especially in the most important aspects of maintaining the health of oral cavities. The answers to the questions in this part are presented in Table 2.

### 3.3. Part C—Patients’ Attitude toward Dental Practice (n = 13)

The answers to the questions contained in this part are presented in Table 3.

### 3.4. Regression Models

Logic regression model was used to evaluate the influence of a person’s characteristics on the probability of eating too much (declaration of eating too much) and on the probability of introducing of cariogenic food during first lockdown. The person’s characteristics included age, gender, region, living with family or alone, having children, education level, and having tested positive or not. Model specifications were selected based on Akaike information criterion (AIC).

#### 3.4.1. Probability of Eating Too Much

Model estimation result is presented in Table 4.

**Figure 1:**
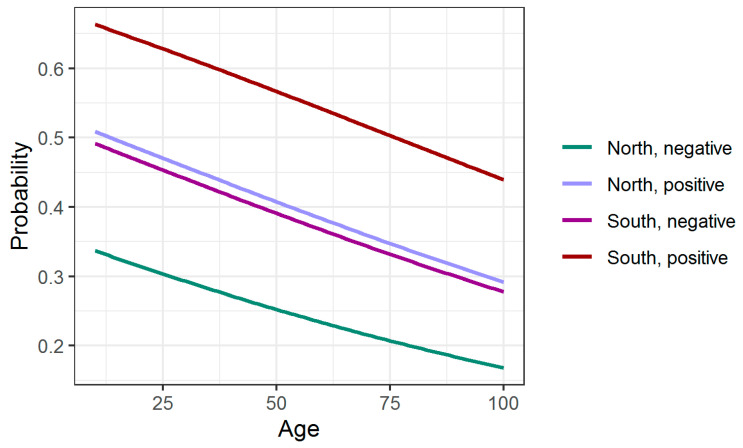
Probability response curves for eating too much.

Living in the south macro region (comparing with north) and having tested positive in the past have statistically significant influence on the probability. Log of the odds ratio increases by 0.643 and 0.712 for persons living in the south region and for persons who have tested positive in the past, respectively. Figure 1 presents the probability response curves for persons living in north/south macro regions and those having tested positive/negative.

#### 3.4.2. Probability of Introducing Cariogenic Food

Model estimation result is presented in Table 5.

**Figure 2:**
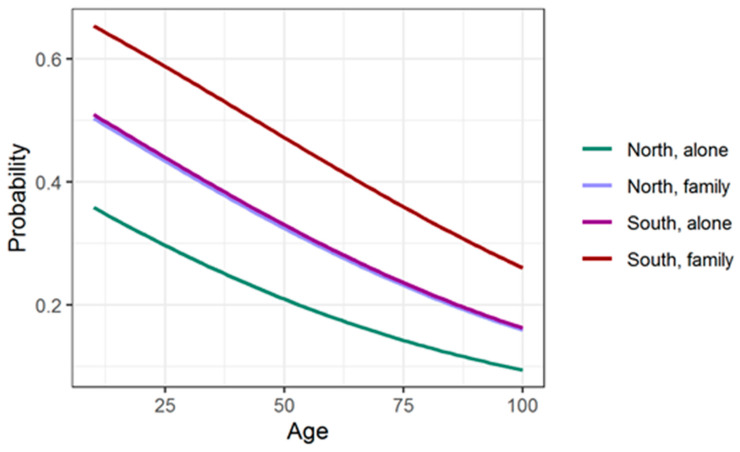
Probability response curves for introducing of cariogenic food. Curves “North, family” and “South, alone” almost coincide.

Age, region, and living with family have statistically significant influence on the probability. The influence of age is negative (elder = less probable), and the influence of living in the center or south macro region (comparing with north) and of living with family is positive. Log of the odds ratio decreases by 0.19 for every 10 years of age; increases by 0.535 or 0.620 for persons living in the center or south, respectively; and increases by 0.595 for persons living in a family. Figure 2 presents the probability response curves for persons living in north/south macro regions and living with family or alone.

## 4. Discussion

This survey, conducted during the months of November and December 2020, enrolled 1135 subjects throughout Italy. The specificity of the organization of this study resulted from the possibility of engaging about 10 dental hygienists throughout the country, all belonging to the Atasio Scientific Association, which allowed for high capillarity in the distribution of enrolled subjects. The main objective of the study was to collect data on the attitudes of patients towards dental procedures in private settings, while the secondary objective was to collect data on lifestyle and the ability to maintain good oral hygiene at home. All the questions collected related to the period of restrictions during the COVID-19 pandemic, which hit Italy particularly hard.

The first wave of the COVID-19 pandemic begun in Italy in early March 2020, with restrictive measures as total lockdown in place until early May 2020. The second wave of the COVID-19 pandemic in Italy continued in November and December 2020, when this study was conducted. There was a curfew from 23:00 throughout Italy, the regions were closed, and it was not allowed to move from the province to the province except for proven health or work reasons. Moreover, school activities at all levels took place remotely; congresses, fairs, and all other events that normally involve gatherings were prohibited. Swimming pools, sports centers, spas, and cinemas were closed, and the population was instructed not to leave their homes unless for serious health or professional reasons, and all who may were called upon to work remotely. From 6 November, 2020, a curfew was applied throughout the country from 10:00 p.m. to 5:00 a.m. the next morning, and movement during this time period was to be only allowed due to the need to work or proven health reasons. The territory of the country was divided into white, orange, and red zones according to the values of the Rt number. In red zones with Rt values above 1.5, where there was a fear of overloading health services, very strict restrictions were in place, with travel bans, restaurants, shops, and malls closing; distance education from the second middle class; and only grocery stores and pharmacies open along with some personal services. These restriction measures were extended for an additional period of 50 days, lasting form 6 January 2021 until 7 July 2021 [10].

At the time of submitting the questionnaire, 64.8% of the respondents said they were negative for COVID-19 (Q9), while in the past, the percentage had risen to 79.5% (Q10). When it was asked if the participants knew someone who has contracted COVID-19, the vast majority say that 69.9% were friends, only 13.3% family members, and 4.2% has been ill themselves. A minority of only 12.6% said they had never known someone who caught the virus (Q39). This answer reflected the epidemic nature of the COVID-19 infection. Moreover, for the answer to question Q33, "What worries you the most?", where a maximum of two answers could be selected, the majority of responses (61.8%) focused on the probability that a family member contracted the virus; while 22.6% said they were concerned about being personally infected with the virus. These responses clearly showed how widespread the spread of the virus was and how fear for a family member was the basis for accepting the restriction measures. A recent work by Ornell F. et al. tried to understand the psychological and psychiatric repercussions of a pandemic and the emotions involved in it, such as fear and anger. The authors underlined that, according to the recent WHO and the U.S. Center for Disease Control and Prevention recommendations, a series of psychosocial and mental health measures during pandemics and large-scale disasters should be implemented over a large scale in populations [11].

In this scenario, the dental office was identified as a place where the risk of infection could rise to its maximum, as in dentistry, face-to-face communication and consistent exposure to body fluids, such as blood and saliva, are frequent and common [3,12,13].

The following questions (Q11 to Q18) were designed to examine the patients’ relationship to dental procedures at the time of the questionnaire administration (November and December 2020) and before the COVID-19 outbreak to try to analyze the difference in choices between the before and after periods.

The analyzed sample consisted of people who, before the emergence of COVID-19, in the vast majority (83.2%) underwent professional oral hygiene once (46.5%) or twice (36.7%) times a year. Only 7.4% of the respondents declared that they had not submitted to a professional oral hygiene session even once a year (Q11). Following the onset of COVID-19, the 7.4% who previously had declared to had not had a single professional oral hygiene session per year increased by 47% (Q12). An increase of 40%+ of people who opted not to have professional oral care after the emergence of COVID-19 was explained by the fear of contracting or infecting their family members. Fifty-three percent of people who decided to undergo oral hygiene treatment anyway during the first and second wave of COVID-19 declared that 38.2% had undergone tartar ablation using ultrasound, 39.6% had manual tartar ablation, and only 3.8% reported whitening and 3.3% treatment of dentin hypersensitivity (Q13). These results are in accordance with the findings of a recent international survey by Campus G et al., where it was shown that access to routine dental care was reduced due to country-specific temporary lockdown periods [14]. The above-mentioned increase in fear of infection among patients may be the most leading factor. With such a reduced frequency of visits, attention should be paid to the lack of profitability of medical activity due to the stable cost of maintaining the dental office. This could have had the obvious effect of diminishing the availability of care in less affluent settings [15].

Participants were asked on a scale of 0 to 10 to rate the extent to which the fear of undergoing dental procedures increased due to the possibility of contracting the COVID-19 virus (Q15) and to what extent this fear caused a change in lifestyle (Q16) and to what extent, as a consequence, the emphasis on home oral hygiene was changed (Q17). Interestingly, 39.1% declared 0 to question 15, but only 9.3% answered 0 to question 16, and more than a third of respondents declared that they had not changed their home oral hygiene habits at all. In addition, questions 19–21 were related to home oral hygiene routines. The vast majority 91.7% of respondents stated that they used a toothbrush (38.4% electric, 53.3% manual), and it is worth emphasizing that 8.3% of respondents declared that they did not use any type of toothbrush (Q19). The use of dental floss accounted for less than half of the sample (46.9%), and it is worrying that more than half, i.e., 53.1%, do not used it. It is worth noting that the data on the use of dental floss are much better than those reported in the literature. In fact, a Delta Dental survey conducted in 2014 showed that only four out of ten Americans floss daily, and 20% of American never floss [16].

Then 45.8% said they did not use any mouthwash, 29.1% used an ozone-based one, and 17.6% used a povidone-iodine one (Q20). Interestingly, ozone-based rinses have well-documented effectiveness in neutralizing most kinds of viruses possibly present in oral cavity [17,18]. This information is in line with the initial data that indicated that subjects paid attention to dental health and that 83.2% received regular checkups and hygiene sessions. In fact, the use of ozone- and iodine-based mouthwashes reflects patients’ access to information that can only be communicated in the professional sphere, emphasizing that they continued to care deeply about oral health and prevention. Probiotics were not used by 86.1% of the respondents, let alone the plaque detector (4.1%) and the tongue cleaner (1.1%) (Q21). Plenty of data exist on oral hygiene during COVID-19 pandemics. For example, a study by Bains KV and Bains R underlined that the oral cavity is portal for the virus’s entry into the human body and that oral hygiene is essential in reducing infection from the oral cavity and thus its transfer to the upper and lower respiratory tract [19]. Moreover, scientific evidence suggests maintaining optimal oral health status minimizes the incidence of hospital-acquired pneumonia [20].

Questions 22 to 25 concerned lifestyle, in particular diet and smoking habits during periods of blockade, in which most people were forced to a more sedentary lifestyle and were exposed to stressful media images.; 64.2% said they abused sweets, 54.2% pizza and bread, and 10.1% meat (Q22 and Q23). The claim of abusing meat means that there is an emerging public awareness of the environmental and health impacts of meat. A total of 32.6% of the sample declared themselves to be non-smokers, and 14.5% smokers; only 11.8% said they smoke more than before COVID-19 (Q24). However, since smokers are a high-risk group for COVID-19 complications, dental hygienists need to find a way to providing effective help in smoking cessation [21]. Almost half of the respondents (47%) stated that they introduced more cariogenic foods into their diet during the lockdown (Q25). These results are in accordance with evidence found in literature. A study by Docimo R et al. on cariogenic risk and COVID-19 lockdown in a pediatric population showed that only 18.6% of the participants had high adherence to a Mediterranean diet, recording an overall increase in sweets consumption and the number of meals. Moreover, Docimo R. et al. showed that also the lifestyle habits before and during lockdown changed significantly, with an overall reduction of the sport activities during and after compared to before [22]. A national Italian survey evaluated eating habits and lifestyle changes in an adult population during COVID-19 lockdown. The study showed that with regards to eating habits and to the hunger/satiety perception, 1214 (34.4%) of the participants declared more appetite. Data on food intake showed an increase of homemade recipes (e.g., sweets, pizza, and bread), cereals, legumes, white meat, and hot beverages consumption. Moreover, 48.6% of the population declared a weight gain perception [23]. Unfortunately, the increase in dietary carcinogenicity along with the increase in weight in a significant part of the society should alarm dental hygienists. All of the above factors significantly contribute to the increased risk of periodontal disease exacerbation as well as to its longer and more difficult treatment in the future [24].

The last part of the questions, Part C, was about patients’ attitudes to dental practices during lockdown, in particular about fears, concerns, experienced feelings, and preventive measures taken by dental offices, which were paid more attention by patients. The vast majority of respondents (86.2%) replied that they had never canceled the dental visit for fear of contracting the COVID-19 virus (Q30). Another 37.2% said they were not worried about having to visit the dentist for dental procedure as surgery, while 55.9% said they were worried to some extent (Q29). A total of 26% declared that they know that the employees of the dental practice/dental clinic they go to were regularly tested for COVID-19, and even 69.5% declared that they do not know whether tests are carried out on employees (Q 35). However, 41.5% said they felt very safe when visiting the dentist (Q34). Infection prevention measures in the dental office that patients paid more attention were (i) the use of personal protective equipment (33%); (ii) sanitations of rooms (19.8%); and (iii) limited access to waiting rooms (19.3%). Of these measures, the one that made patients feel safe more was the sanitation of the environments (32.1%) and the use of personal protective equipment (28.6%) (Q27 and 28).

The present survey evaluated also the psychological and psychiatric repercussions of the pandemic among the dental patients: 75% of respondents declared that they feel tired, more than half declared that they did not feel joy in everyday activities, half declared that they had difficulties concentrating and making difficult decisions and felt unhappy and scared (Q32). The need for empathy, interpersonal contact, and communication was reflected in the answer to question 37, where 73.2% declared that despite the social distance and the need for health care workers and dentists to wear very bulky personal protective equipment that cover the face, they still considered communication with the doctor/hygienist to be easy and effective. A total of 23.3% of respondents stated that they would like to stay in touch with their dentist through telemedicine services, 16.4% through telephone consultations, and 24.4% would like to receive videos of home hygiene instructions of oral cavity (Q39). Interestingly, as research shows, the vast majority of dental practitioners are not aware of how to utilize proper software for telemedicine and do not know how a conversation with a patient should proceed during teleconsultation in order to effectively check the condition of the oral cavity and provide comprehensively appropriate advice [25].

### 4.1. Limitations

Given the large number of patients enrolled in this observational survey study, it is possible to highlight the national setting of the study as a possible limitation. Certainly, if it was possible to organize a research group in Europe, the analyzed data could provide a broader representation of the studied phenomenon.

### 4.2. Recommendations

The results of this study emphasize that the character of the dentist is fundamental to the promotion of oral health and a healthy lifestyle. The role of dentistry in the daily implementation of primary and secondary prevention should be strengthened, and general practitioners (GPs) should be aware of their potential in this sense.

Continuing education programs for GPs on healthy lifestyles should be implemented, especially in the COVID-19 scenario.

Finally, it is to be hoped that technologies such as telemedicine will be further developed and disseminated in order to ensure contact between the doctor and the patient, emphasizing the importance of this possibility especially for patients with special needs but also for the sustainable development of dentistry [26].

## 5. Conclusions

COVID-19 has changed patients’ approach to dental procedures. Most of the people interviewed lived in families, and their greatest fear was infecting a family member. Restrictive measures forced people to stay at home longer, which led to an increase in consumption of various types of food, including cariogenic foods. People said they felt safe when they went to the dentist, but they also paid special attention to measures to prevent contagion. Despite the social distancing and PPE worn by healthcare professionals in dentists’ offices, doctor–patient communication was important. Finally, among the measures that should be introduced in similar situations in the future, people wanted telemedicine, a phone recall, and the possibility of using video clips for home oral care instructions.

## Figures and Tables

**Table 1:** Characteristics of the respondents.

Questionnaire Part A: Characteristics of Respondents		
Q1: Age	14–20	34 (3%)
	20–40	588 (51.8%)
	40–60	434 (38.2%)
	60–80	79 (7%)
Q2: Gender	Female	782 (68.9%)
	Male	353 (31.1%)
Q3: Area of geographic origin	North Italy	233 (20.6%)
	Center Italy	614 (54.1%)
	South Italy	288 (25.3%)
Q5: Civil status	Married	725 (63.9%)
	Single	294 (25.9%)
	In separation	97 (8.6%)
	Widow(er)	19 (1.6%)
Q6: Do you have any children?	Yes	539 (45.8%)
	No	596 (54.2%)
Q7: Do you live with your family?	Yes	910 (80.2%)
	No	225 (19.8%)
Q8: Level of education	Basic	68 (6%)
	Secondary	534 (47%)
	Higher	533 (46.7%)
Q9: In this moment, you are positive for COVID-19?	Yes	16 (1.4%)
	No	736 (64.8%)
	I don’t know	383 (33.7%)
Q10: Have you ever tested positive for COVID-19 in the past?	Yes	39 (3.4%)
	No	903 (79.5%)
	I don’t know	193 (17.1%)

**Table 2:** Questionnaire Part B—respondents’ attitude toward oral health selfcare and lifestyle.

Questionnaire Part B: Respondents’ Attitude toward Oral Health Selfcare and Lifestyle				
Q11: How many times a year do you have an oral hygiene session (before COVID-19)?	0	84 (7.4%)		
	1	527 (46.5%)		
	2	416 (36.7%)		
	3	83 (7.3%)		
	4	22 (2%)		
Q12: Did you have had an oral hygiene session from March 2020 to November 2020?	Yes	602 (53%)		
	No	533 (47%)		
Q13: What type of therapy have you been subjected to?	Ultrasound tartar ablation	433 (38.2%)		
	Manual tartar ablation	449 (39.6%)		
	Whitening	43 (3.8%)		
	Dentin hypersensitivity treatment	37 (3.3%)		
Q14: When was the last time you went to the dentist?	Don’t remember	47 (4.1%)		
	Last 3 months	471 (41.5%)		
	Last 6 months	230 (20.3%)		
	Last year	267 (23.5%)		
	Last 2 years	120 (10.6%)		
Q15: From March 2020, on a scale of 0 to 10, how much has your fear of undergoing dental procedures increased (due to COVID-19 contagion)?		Q15	Q16	Q17
	0	444 (39.1%)	106 (9.3%)	368 (32.4%)
	1	68 (6%)	42 (3.7%)	53 (4.7%)
	2	104 (9.2%)	64 (5.6%)	47 (4.1%)
Q16: How much does the fear of contagion affect your lifestyle (scale from 0 to 10)?	3	84 (7.4%)	94 (8.3%)	64 (5.6%)
	4	53 (4.7%)	93 (8.2%)	49 (4.3%)
	5	93 (8.2%)	173 (15.2%)	129 (11.4%)
	6	84 (7.4%)	126 (11.1%)	85 (7.5%)
Q17: How much has your attention changed towards home oral hygiene maneuvers (scale from 0 to 10)?	7	66 (5.8%)	121 (10.7%)	90 (8%)
	8	78 (6.9%)	173 (15.2%)	107 (9.4%)
	9	24 (2.1%)	82 (7.2%)	51 (4.5%)
	10	37 (3.3%)	65 (5.7%)	99 (8.1%)
Q18: How worried were you about contracting the coronavirus during a dental hygiene session?	Not at all	389 (34.3%)		
	A little	484 (42.6%)		
	Somewhat	163 (14.4%)		
	A lot	64 (5.6%)		
	Very	18 (1.6%)		
	I don’t know	16 (1.4%)		
Q19: Which aids did you use for at-home oral hygiene during the first lockdown (March–May 2020). More than one answer was possible.	Manual brush	605 (53.3%)		
	Electric brush	435 (38.4%)		
	Floss	532 (46.9%)		
	Tongue cleaner	12 (1.1%)		
	Plaque detector	47 (4.1%)		
	I don’t know	64 (5.6%)		
Q20: Did you use rinse at home during the first lockdown?	No	520 (45.8%)		
	Chlorhexidine-based	54 (4.8%)		
	Ozone-based	330 (29.1%)		
	Iodopovidone-based	200 (17.6%)		
	Hydrogen peroxide-based	5 (0.4%)		
	Commercial mouthwash	26 (2.3%)		
Q21: Did you use probiotics during the first lockdown?	Yes	158 (13.9%)		
	No	977 (86.1%)		
Q22: Do you think you have eaten too much of any type of food during the lockdown?	Yes	442 (38.9%)		
	No	693 (61.1%)		
Q23: If you have abused any food, write which one.	Sweets	729 (64.2%)		
	Bread, pizza	615 (54.2%)		
	Wine	106 (9.3%)		
	Meat	115 (10.1%)		
Q24: Do you think you smoked more during the lockdown than before?	Yes	134 (11.8%)		
	No	364 (32.1%)		
	I have never smoked	370 (32.6%)		
	Now I do not smoke	165 (14.5%)		
	More than 10 a day	47 (4.1%		
	Less than 10 a day	55 (4.8%)		
Q25: Did you implement cariogenic foods during the lockdown (pasta, bread, sugars, sweets, snacks, chocolate)?	Yes	533 (47%)		
	No	602 (53%)		

**Table 3:** Questionnaire Part C—Respondents’ Attitude toward Dental Practice.

Questionnaire Part C: Respondents’ Attitude toward Dental Practice					
Q26: Which coronavirus prevention measures do you pay attention to most when you go to the dentist? Doing which of the previously listed procedures make you feel safer?		Q26	Q27		
	Sanitation of environments	225 (19.8%)	364 (32.1%)		
	Room ventilation	40 (3.5%)	69 (6.1%)		
	Temperature control	80 (7%)	33 (2.9%)		
	Use of protective equipment	375 (33%)	325 (28.6%)		
	Limitation of entries to the waiting area	219 (19.3%)	226 (19.9%)		
	Providing patients with masks and disinfectants	49 (4.3%)	43 (3.8%)		
	Telephone Pre-visit medical history check-up	78 (6.9%)	44 (3.9%)		
	All answers	15 (1.3%)	9 (0.8%)		
Q28: Are you worried about going to visit a dentist to have a dental procedure?	Not at all	422 (37.2%)			
	A little	493 (43.4%)			
	Somewhat	142 (12.5%)			
	A lot	44 (3.9%)			
	Very	12 (1.1%)			
	Don’t visit a dentist	9 (0.8%)			
	Don’t know	12 (1.1%)			
Q29: Have you ever postponed the appointment for fear of becoming infected with COVID-19?	Yes	129 (11.4%)			
	No	978 (86.2%)			
	I haven’t visited a dentist in this period	28 (2.5%)			
		I don’t feel it	I feel it lightly	I feel it in a moderate way	I feel it in an extreme way
Q30: What emotions stir in you to think about the coronavirus?	Fear	269 (23.7%)	494 (43.5%)	343 (30.2%)	30 (2.6%)
	Anxiety	300 (26.4%)	432 (38.1%)	339 (29.9%)	64 (5.6%)
	Worry	125 (11%)	502 (44.2%)	446 (39.3%)	62 (5.5%)
	Sadness	287 (25.3%)	384 (33.8%)	353 (31.1%)	111 (9.8%)
	Anger	434 (38.2%)	280 (24.7%)	292 (25.7%)	129 (11.4%)
	Powerlessness	292 (25.7%)	312 (27.5%)	352 (31%)	179 (15.8%)
	Distress	440 (38.8%)	377 (33.2%)	254 (22.4%)	64 (5.6%)
	Aggression	804 (70.8%)	232 (20.4%)	77 (6.8%)	22 (1.9%)
	Shame	907 (79.9%)	154 (13.6%)	67 (5.9%)	7 (0.6%)
	Disbelief	518 (45.6%)	329 (29%)	197 (17.4%)	91 (8%)
	Isolation	383 (33.7%)	381 (33.6%)	284 (25%)	89 (7.8%)
		Never	Sometimes	Often	Always
Q31: Please indicate how often each of the following problems have bothered you in the past two weeks (Spitzer et al., 2006) [9]	Feeling more nervous anxious tense than usual	262 (23.1%)	648 (57.1%)	163 (14.4%)	62 (5.5%)
	Not being able to stop worrying or keep worries under control	438 (38.6%)	501 (44.1%)	154 (13.6%)	42 (3.7%)
	Worrying too much about various things	339 (29.9%)	516 (45.5%)	214 (18.9%)	66 (5.8%)
	Having trouble relaxing and sleeping well	394 (34.7%)	484 (42.6%)	190 (16.7%)	68 (6.0%)
	Difficulty concentrating	472 (41.6%)	470 (41.4%)	144 (12.7%)	49 (4.3%)
	I find it difficult to make decisions	579 (51.0%)	392 (34.5%)	126 (11.1%)	38 (3.3%)
	Difficulty enjoying daily activities	395 (34.8%)	431 (38.0%)	221 (19.5%)	89 (7.8%)
	Tired out	292 (25.7%)	487 (42.9%)	263 (23.2%)	93 (8.2%)
	Getting scared easily	650 (57.3%)	350 (30.8%)	96 (8.5%)	37 (3.3%)
	Feeling unhappy	482 (42.5%)	451 (39.7%)	148 (13.0%)	54 (4.8%)
	Trouble thinking straight	692 (61%)	339 (29.9%)	79 (7.0%)	24 (2.1%)
	Being so restless that it is difficult to sit still	787 (69.3%)	241 (21.2%)	86 (7.6%)	22 (1.9%)
	Easily annoyed or irritated	450 (39.6%)	482 (42.5%)	148 (13.0%)	56 (4.9%)
	Being afraid that something terrible might happen	499 (44.0%)	442 (38.9%)	136 (12.0%)	59 (5.2%)
Q32: What worries you the most? Choose 2 most relevant responses.	Not knowing when the emergency will end	644 (56.7%)			
	Possibility of sickness of a family member	701 (61.8%)			
	Reduced economic availability	205 (18.1%)			
	Possibility of losing the future, planning	299 (26.3%)			
	Possibility of getting sick	256 (22.6%)			
Q33: Do you feel safe visiting your dentist?	No	111 (9.8%)			
	Somewhat	512 (45.1%)			
	Very	471 (41.5%)			
	Don’t know	41 (3.6%)			
Q34: At the dentist’s office you go to, are the staff tested for COVID-19?	Yes	295 (26%)			
	No	1 (0,09%)			
	I don’t know	789 (69.5%)			
	Never	50 (4.4%)			
Q35: What type of instrument does your hygienist use during the COVID-19 pandemic? (Manual or mechanic)	Manual	268 (23.6%)			
	Mechanic	217 (19.1%)			
	Both, but mostly mechanic	256 (22.6%)			
	Both, but mostly manual	240 (21.2%)			
Q36: Knowing that in this pandemic situation in which social distancing is fundamental it is also essential to wear very personal protective equipment that covers almost the entire body and face, communication with your dentist/hygienist is:	Easy	831 (73.2%)			
	Complicated	235 (20.7%)			
Q37: Which of these additional treatments would you like to be able to use during this period?	Telemedicine	264 (23.3%)			
	Telephone consultation	186 (16.4%)			
	Home hygiene instructions video	277 (24.4%)			
	I don’t know	407 (35.9%)			
Q38: Do you know anyone who has contracted the coronavirus?	Nobody	143 (12.6%)			
	Myself	48 (4.2%)			
	Family members	151 (13.3%)			
	Friends	793 (69.9%)			

**Table 4:** The model estimation for the probability of eating too much during first lockdown.

Variable	Estimate	Std. Error	*z*-Value	*p*-Value
Intercept	−0.574	0.328	−1.751	0.080
Age	−0.010	0.007	−1.426	0.154
Male gender	−0.320	0.178	−1.796	0.072
Center of Italy	0.203	0.220	0.925	0.355
South of Italy	0.643	0.248	2.587	**0.010**
Having children	0.353	0.187	1.888	0.059
Being positive to COVID-19 in the past	0.712	0.346	2.057	**0.040**
Model characteristics	AIC: 900.28			
	McFadden’s R^2^ = 0.019			

**Table 5:** The model estimation for probability of introducing cariogenic food during first lockdown.

Variable	Estimate	Std. Error	z-Value	*p*-Value
Intercept	−0.395	0.349	−1.133	0.257
Age	−0.019	0.006	−3.030	**0.002**
Center of Italy	0.535	0.214	2.503	**0.012**
South of Italy	0.620	0.243	2.555	**0.011**
Live with family	0.595	0.205	2.903	**0.004**
Model characteristics	AIC: 935.16			
	McFadden’s R^2^ = 0.031			

## Data Availability

All the raw data are available from corresponding author on a reasonable request.
